# Effect of *Nigella sativa* oil on various clinical and biochemical parameters of insulin resistance syndrome

**DOI:** 10.4103/0973-3930.41980

**Published:** 2008

**Authors:** Ahmad Najmi, Mohammad Nasiruddin, Rahat Ali Khan, Shahzad F. Haque

**Affiliations:** Department of Pharmacology, J. N. Medical College, Aligarh Muslim University, Aligarh, India; 1 Department of Medicine, J. N. Medical College, Aligarh Muslim University, Aligarh, India

**Keywords:** Insulin resistance syndrome, *Nigella sativa*

## Abstract

**AIM:**

The seeds of the *Nigella sativa* plant have been used to promote health and fight disease for centuries, especially in the Middle East and in Southeast Asia. This plant has been a focus of much research. This clinical study was undertaken to know the adjuvant effect of *N. sativa* oil on various clinical and biochemical parameters of the insulin resistance syndrome.

**MATERIALS AND METHODS:**

This prospective study was conducted at a tertiary health care center in North India. After confirmation of diagnosis, 60 patients who fulfilled the inclusion and exclusion criteria were enrolled in this study. Written informed consent was taken from all the patients enrolled. Approval from the institutional ethical committee was also obtained. The patients were divided into two groups of 30 each. In group I (the standard group), patients were advised tablet atorvastatin 10 mg once a day and tablet metformin 500 mg twice a day for a period of 6 weeks. In group II (the *N. sativa* group), the patients were advised tablet atorvastatin 10 mg once a day, tablet metformin 500 mg twice a day, and *N. sativa* oil 2.5 ml twice daily for a period of 6 weeks. Fasting and postprandial blood glucose, fasting lipid profile, and waist circumference were recorded before therapy and after completion of therapy.

**RESULT:**

The treatment group showed significant (*P* < 0.05) improvement with reference to total cholesterol, low density lipoprotein cholesterol (LDL-C), and fasting blood glucose (*P* < 0.05).

**CONCLUSION:**

*N. sativa* oil was found to be effective as an add-on therapy in patients of insulin resistance syndrome. *N. sativa* oil has a significant activity in diabetic and dyslipidemic patients.

## Introduction

The seeds of the *Nigella sativa* plant have been used to promote health and fight disease for centuries, especially in the Middle East and in Southeast Asia. In South Asia, it is called *kalonji*; its Arabic name is *habat-ul-sauda* and in English it is known as black cumin. This plant has been a focus of much research. It has several traditional uses and consequently has been extensively studied for its chemical constituents and biological activities. A lot of animal studies have already been done to identify the various activities of *N. sativa* oil on different components of the metabolic syndrome, for example blood glucose[1] and blood pressure.[2] However, no clinical studies have been done in patients of the metabolic syndrome. This clinical study was undertaken to find out the adjuvant effects of *N. sativa* oil on various clinical and biochemical parameters of the metabolic syndrome.

## Materials and Methods

This prospective study was carried out on patients who were attending the OPD of the Department of Medicine and Nephrology, J.N. Medical College, from April 2006 to March 2007. After confirming the diagnosis, 60 patients (50 males, 10 females) who fulfilled the inclusion and exclusion criteria were enrolled for this study. Written informed consent was taken from all the patients. Approval from the institutional ethical committee was also obtained.

### Inclusion criteria

1. Abdominal obesity: Waist circumference
  >102 cm for males
  >88 cm for females
2. Serum triglycerides >150 mg%
3. Serum high density lipoprotein (HDL) <50 mg%
4. Blood pressure >140/90 mmHg
5. Fasting blood sugar >110 mg%

The metabolic syndrome was diagnosed if three or more of the above criteria were present, whether the patient was or was not on treatment.

### Exclusion criteria

Pregnancy, type I diabetes mellitus, impaired liver function test, patients of chronic renal disease, primary dyslipidemia, myopathy.

All the patients were thoroughly examined and a detailed history was taken.

The baseline parameters measured in our study were the following:

1. Waist circumference
2. Fasting and postprandial blood glucose
3. Lipid profile: total cholesterol (TC), low density lipoprotein cholesterol (LDL-C), high density lipoprotein cholesterol (HDL-C), and triglyceride (TG)

The values of the baseline parameters were recorded before therapy and after completion of therapy.

#### *N. sativa* oil

*N. sativa* oil (Kalonji oil, Mohammedia Products, Red Hills, Nampally, Hyderabad) was procured from the local market at Aligarh. As per the manufacturer's information, it had been prepared by steam distillation at Hyderabad, Andhra Pradesh, India.

### Study groups

The 60 patients were divided into two groups on the basis of the clinical profile and the therapeutic intervention.

**Group 1** (the standard group; *n* = 30): Patients with obesity, diabetes, and dyslipidemia; they were given the standard regimen.

**Group 2** (*N. sativa* group; *n* = 3): Patients with obesity, diabetes, and dyslipidemia; they were given the standard regimen plus *N. sativa* oil 2.5 ml twice daily per orally for a period of 6 weeks.

**Standard regimen:** Tablet metformin 500 mg twice a day and tablet atorvastatin 10 mg once a day.

### Statistical analysis

The differences between the percentage improvements in each parameter in the two groups was compared by applying the unpaired *t*-test using the SPSS software [Table 1].

**Table 1:** Clinical and biochemical characteristics of controls (Group 1) and treated subjects (Group 2)

Parameter	Group	No. of patients (*n*)	Mean percentage improvement	Standard deviation
Abdominal circumference	1	30	0.1668	0.6406
	2	30	0.5228	0.9816
Fasting blood sugar	1	30	18.4637	6.7724
	2	30	29.2392	6.0937
Postprandial blood glucose	1	30	19.8745	6.2160
	2	30	23.3879	8.5426
Total cholesterol	1	30	16.9249	6.2515
	2	30	26.8714	6.7530
Triglyceride	1	30	14.1829	0.0090
	2	30	12.0269	0.5474
High density lipoprotein	1	30	13.9678	3.1939
	2	30	15.8937	2.1528
Low-density lipoprotein	1	30	15.9471	5.7503
	2	30	23.8898	7.2984

## Results

The results are presented in Table 2, which is self-explanatory.

**Table 2:** Comparison of *N. sativa* oil-treated subjects with controls

	Group 1 (control subjects)	Group 2 (treated subjects)	*P*
Reduction in abdominal circumference (cm)	05.228 ± 0.9816	0.5228 ± 0.9816
Reduction in FBG (mg/dl)	18.4637 ± 6.772	29.2392 ± 6.0937	0.000
Reduction in PPBG (mg/dl)	19.874 ± 6.2160	23.3879 ± 8.5426
Reduction in total cholesterol (mg/dl)	16.9249 ± 6.2515	26.8714 ± 6.7530	0.000
Reduction in TG (mg/dl)	14.1829 ± 0.0090	12.0269 ± 0.5474
Increases in HDL-C (mg/dl)	13.9678 ± 3.1939	15.8937 ± 2.1528
Reduction in LDL-C (mg/dl)	15.947 ± 5.7503	23.8898 ± 7.2984	0.012

## Discussion

Till date, many animal studies have been done on the various parameters of the metabolic syndrome and most studies have reported a favorable effect of *N. sativa* on the various components of this syndrome. Waist circumference[3] has a very crucial role in the detection and diagnosis of the metabolic syndrome. Overweight and obesity are associated with insulin resistance and the metabolic syndrome, but the presence of abdominal obesity is more highly correlated with the metabolic risk factors than is an elevated BMI. Therefore, the simple measure of waist circumference is recommended to identify the body weight component of the metabolic syndrome. Some male patients can develop multiple metabolic risk factors when the waist circumference is only marginally increased, e.g., 94-102 cm (37-39 inches). The insulin resistance in such patients may have a strong genetic component. These patients should benefit from changes in life habits, similarly to men with categorical increases in waist circumference.

In our study, reduction in abdominal circumference in the *N. sativa* group was more than seen in the standard group but this difference was not statistically significant.

Fasting and postprandial blood sugar is a very important parameter for the diagnosis of the metabolic syndrome. Near-normal or improved glycemic control[4] has been shown to significantly diminish the risk of microvascular complications in patients with type 2 diabetes mellitus and that is why this criterion is included in most of the definitions of the metabolic syndrome. In our study, the cut-off point for fasting blood glucose was 110 mg%, which is the value recommended in the ATP III criteria[3] for the diagnosis of metabolic syndrome. Reduction in fasting blood sugar in the *N. sativa* group was significantly higher (*P* = 0.000) than in the standard group. Postprandial blood sugar is also an important measurement to assess the glycemic control. The percentage reduction in postprandial blood glucose also was more in *N. sativa* group as compared to the standard group, but this difference was not significant. In our study, the reduction in total cholesterol in the *N. sativa* group was significantly more (*P* = 0.000) than in the standard group. Previous studies[5] also have reported a cholesterol-lowering effect of *N. sativa* oil. Previous research workers suggested various mechanisms by which *N. sativa* may be effective in lowering the cholesterol level and in improving the lipid profile. For example, it was postulated that the seeds may either inhibit *de novo* cholesterol synthesis[6] or stimulate bile acid excretion.[7] It is well known that both effects would lead to a decrease in serum cholesterol. Further research is necessary to identify the mode of action of black cumin seeds. Another mechanism that was proposed is that *N. sativa* increases the production of LDL receptors.[8] The presence of various unsaturated fatty acids,[10] such as arachidonic, eicosadienoic, linoleic, linolenic, oleic, and almitoleic acid, may be responsible for the improvement in the lipid profile.

In our study, the cut-off value for triglyceride was 150 mg%, which is as per the ATP III guidelines. Reduction in triglyceride in the *N. sativa* group was less than in the standard group. Previous studies[9] also have reported that *N. sativa* oil has no significant effect on triglyceride level. In our study, the cut-off point for HDL was 50 mg%. Although the normal HDL level varies slightly according to sex, we took a single cut-off point of 50 mg% for both sexes for the purpose of simplicity. Increase in HDL in the *N. sativa* group was more than in the standard group, but this difference was not statistically significant. Previous studies have reported varying results with regard to the effect of *N. sativa* oil on HDL levels. Some studies[9] reported that HDL was increased, while others[10] reported that *N. sativa* had no effect on HDL. Reduction in LDL in the *N. sativa* group was more than in the standard group and this reduction was significant (*P* = 0.012). Previous research workers[9] also have reported the similar results from various animal studies.

*N. sativa* has a beneficial effect on fasting blood glucose, total cholesterol, and LDL cholesterol. More research is required to find out the various mechanisms by which *N. sativa* acts on the various components of the metabolic syndrome. *N. sativa* is a remedy that may prove to be useful in the prevention and treatment of the insulin resistance syndrome.

## References

[1] Bamosa AO (1997). Effect of oral ingestion of *Nigella sativa* seeds in some blood parameters. Saudi Pharm J.

[2] Aqel M (1992). The relaxing effect of the volatile oil of *Nigella sativa* seeds on vascular smooth muscle. Dirasat Series B Pure and Applied Sciences.

[3] National Institutes of Health (2001). Third Report of the National Cholesterol Education Program Expert Panel on Detection, Evaluation and Treatment of High Blood Cholesterol in Adults (Adult Treatment Panel III). Executive Summary.

[4] (1998). Tests of glycemia in diabetes. Diabetes Care.

[5] (1993). Second report of the Expert Panel on Detection, Evaluation and Treatment of High Blood Cholesterol in Adults (adult treatment panel II).

[6] Bamosa AO, Ali BA, al-Hawsawi ZA (2002). The effect of thymoquinone on blood lipids in rats. Indian J Physiol Pharmacol.

[7] Bamosa AO (1997). Effect of oral ingestion of *Nigella sativa* seeds in some blood parameters. Saudi Pharm J.

[8] Beynen AC, MB Katan, Van Zutphen LF (1987). Hypo- and hyperresponders: Individual differences in the response of serum cholesterol concentration to changes in diet. Adv Lipid Res.

[9] el-Dakhakhny M (1965). Studies on the Egyptian *Nigella sativa* L Part-IV: Some pharmacological properties of the seeds active principle in comparison to its dihydrocompound and its polymer. Arzneimittelforschung.

[10] Gad AM, El-Dakhakhany M, Hassan MM (1963). Studies on the chemical constitution of Egyptian *Nigella sativa* L oil. Planta Med.

